# Topical ruxolitinib in combination with oral minoxidil results in hair regrowth in a patient with central centrifugal cicatricial alopecia and concomitant traction alopecia

**DOI:** 10.1016/j.jdcr.2025.04.010

**Published:** 2025-04-24

**Authors:** Gabriela Soto-Canetti, Hassan Hamade, Benjamin Ungar, Jordan Talia

**Affiliations:** Department of Dermatology, Icahn School of Medicine at Mount Sinai, New York, New York

**Keywords:** central centrifugal cicatricial alopecia, JAK inhibitors, ruxolitinib, skin of color, traction alopecia

## Introduction

Central centrifugal cicatricial alopecia (CCCA) is a lymphocyte-predominant scarring disorder of the scalp characterized by hair loss at the vertex or crown that expands centrifugally. This disorder disproportionately affects adult women of African descent, with an estimated prevalence of 2.5% to 5.7%.^1^ CCCA can significantly impact patients’ quality of life, especially when traditional treatment proves ineffective. First-line therapy for CCCA includes topical or intralesional corticosteroids and oral tetracyclines to target follicular inflammation.^1^ Calcineurin inhibitors and minoxidil are used as adjuvant therapy, with hydroxychloroquine, mycophenolate mofetil, or cyclosporine reserved for refractory disease.^1^ Advanced cases of CCCA present a challenge due to the limited availability of effective systemic agents and the risks of immunosuppression associated with traditional systemic treatments. Although the pathogenesis of CCCA remains unclear, recent research implicates the Janus kinase (JAK)-signal transducer and activator of transcription (STAT) pathway.^2^ Several reports have demonstrated the successful use of oral JAK inhibitors (JAKi) in treating cicatricial alopecias.^3^^,^^4^ We present the case of a 55-year-old woman with CCCA and traction alopecia (TA) who demonstrated significant hair regrowth after 6 months of treatment with topical ruxolitinib, a JAK1/2 inhibitor.

## Case presentation

**Fig 1:**
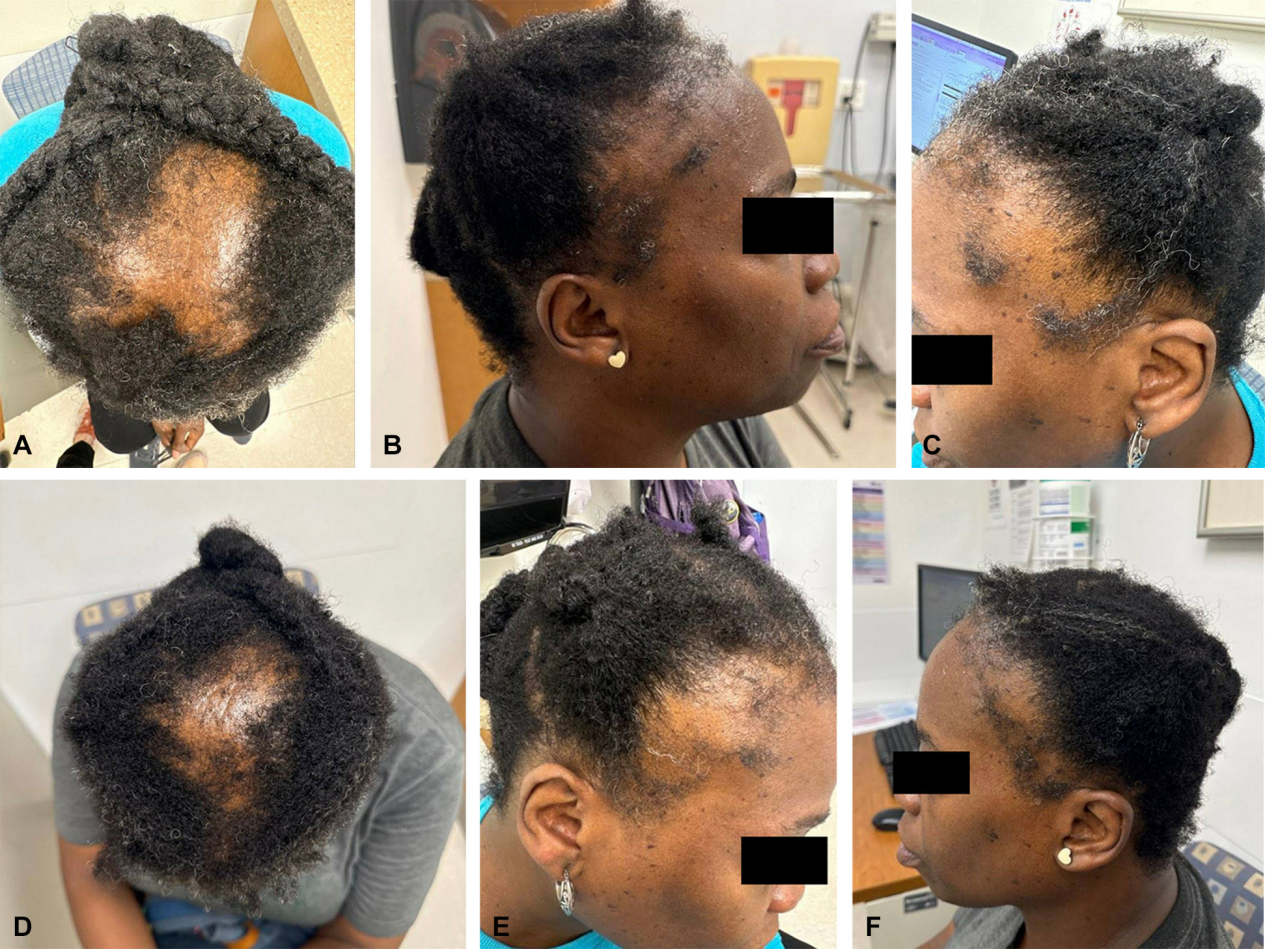
Physical examination reveals areas of decreased hair density on scalp vertex (**A**) and bilateral temporal scalp (**B** and **C**). Results after 6 months of topical ruxolitinib show significant hair regrowth on vertex (**D**) and bilateral temporal scalp (**E** and **F**).

A 55-year-old female presented in February 2023 with complaints of hair loss for several years. She reported using topical minoxidil and oral spironolactone 50 mg daily due to a history of polycystic ovarian syndrome without response. Physical examination revealed hair loss on the vertex and frontal scalp with a positive fringe sign (Fig 1, *A-E*). She was diagnosed with CCCA and TA and treated with oral minoxidil 2.5 mg daily. She was also continued on spironolactone by endocrinology for hair loss that was favored to be related to polycystic ovarian syndrome. Two months later, there was minimal improvement in hair loss, and she was treated with fluocinonide 0.05% solution daily for 2 weeks alongside oral minoxidil. Triamcinolone 0.1% lotion was added a month later due to the patient’s perceived lack of response to treatment. In February 2024, the patient presented to our clinic with little improvement in hair loss after over a year on this treatment regimen and was prescribed ruxolitinib 1.5% cream twice daily in combination with oral minoxidil. Spironolactone, fluocinonide, and triamcinolone were discontinued. Due to the presence of a TA component, she was also counseled on hair care practices to reduce hair loss, including avoiding tight hairstyles, excessive heat styling, and trauma to the scalp. After 6 months of therapy, there was significant improvement in hair loss, with regrowth in both the vertex and bilateral temporal regions of TA (Fig 1, *B-F*) and no reported adverse effects from ruxolitinib. We attribute our patient’s hair regrowth primarily to the use of topical ruxolitinib, as our patient had been on oral minoxidil for the prior 1.5 years.

## Discussion

Currently, no Food and Drug Administration-approved therapies exist for cicatricial alopecias. Recent studies show effective treatment of CCCA and other cicatricial alopecias by JAKi.^3^^,^^4^ The JAK/STAT pathway is hypothesized to play a significant role in the pathophysiology of CCCA.^2^ A study of CCCA specimens revealed increased numbers of perifollicular lymphocytes with phosphorylated STAT3 compared to androgenetic alopecia controls.^2^ STAT3 activation promotes T helper 17 cell activation and proliferation, producing pro-inflammatory cytokines that may be implicated in the perifollicular inflammation and fibrosis in CCCA.^2^ JAKi have recently been utilized as treatment for alopecia areata (AA), leading to the first Food and Drug Administration-approved biologic agent for this condition.^5^ However, large-scale data on JAKi therapy for cicatricial alopecias, especially CCCA, remains minimal. Individual clinical reports of JAKi use in cicatricial alopecias provide a favorable profile for future use of this treatment modality.^3^^,^^4^ A phase 2a study evaluating the efficacy of oral dual JAK1/tyrosine kinase 2 inhibitor brepocitinib in cicatricial alopecias found significant downregulation of inflammatory biomarkers, including CCL5, and demonstrated a favorable safety profile.^6^ To the best of our knowledge, this is the first report showing the effective use of topical ruxolitinib for treating CCCA, as well as TA. Specifically, the use of topical JAKi offers a safer treatment alternative for patients with scarring alopecia, alleviating common concerns about immunosuppression associated with oral JAKi. Notably, a recent clinical trial involving topical ruxolitinib for AA did not result in a significant difference in hair regrowth in the treatment arm.^7^ We hypothesize that, unlike in AA, inflammation in CCCA occurs at the inner root sheath of the lower infundibulum of the hair follicle, a more superficial location, and thus JAKi may be more effective in treating this particular inflammatory process. Our patient demonstrated significant hair regrowth after a 6-month course of topical ruxolitinib, having previously failed treatment with oral minoxidil and topical corticosteroids. We attribute her hair growth mainly to topical ruxolitinib, though we acknowledge the concurrent use of oral minoxidil. While minoxidil may have synergistically influenced our patient’s hair regrowth, it is unlikely that her late-stage TA, as evidenced by the fringe sign, would have responded to oral minoxidil monotherapy due to widespread inflammation and scarring of the hair follicles.^8^^,^^9^ A single-center review of the fringe sign in TA patients found that 57% of samples demonstrated sparse lymphocytic inflammation.^9^ Notably, topical and intralesional corticosteroids, as well as oral antibiotics, have been utilized successfully to treat early-stage TA, highlighting an inflammatory aspect of this condition that can be targeted by topical JAKi.^9^ Another study found increased hair growth along the frontotemporal hairline with concurrent intralesional triamcinolone and topical minoxidil, and one patient showed similar results with intralesional triamcinolone alone.^10^ Furthermore, while TA was also present in the frontal scalp, we cannot rule out the possibility of concomitant CCCA in that area, as TA has been suggested as an aggravating factor for CCCA.^1^ The positive results in our patient underscore the need for further peer-reviewed studies to characterize the effect of topical JAKi like ruxolitinib on scarring alopecias such as CCCA and TA with or without concomittant minoxidil. These findings suggest topical JAKi may serve as a promising therapeutic option for patients with CCCA and/or TA.

## Conflicts of interest

Dr Talia has served as a consultant for AbbVie, Arcutis Biotherapeutics, Bristol Myers Squibb, Calliditas Therapeutics, Johnson & Johnson, LEO Pharma, Primus Pharmaceuticals, Sanofi Genzyme, Stifel Financial, and UCB; and has served as an investigator for LEO Pharma and Sanofi. Dr Ungar is an employee of Mount Sinai and has received research funds (grants paid to the institution) from Bristol Myers Squibb, Incyte, Rapt Therapeutics, Pfizer, and Sanofi; and is also a consultant for Arcutis Biotherapeutics, Bristol Myers Squibb, Botanix Pharmaceuticals, Castle Biosciences, Fresenius Kabi, Galderma, Janssen, Lilly, Pfizer, Primus Pharmaceuticals, Sanofi, and UCB. Drs Hamade and Soto-Canetti have no conflicts of interest to disclose.
